# Structural Characterization of the Xi Class Glutathione Transferase From the Haloalkaliphilic Archaeon *Natrialba magadii*

**DOI:** 10.3389/fmicb.2019.00009

**Published:** 2019-01-18

**Authors:** Adele Di Matteo, Luca Federici, Michele Masulli, Erminia Carletti, Daniele Santorelli, Jennifer Cassidy, Francesca Paradisi, Carmine Di Ilio, Nerino Allocati

**Affiliations:** ^1^Institute of Molecular Biology and Pathology, CNR, Rome, Italy; ^2^Department of Medical, Oral and Biotechnological Sciences, “G. d’Annunzio” University of Chieti-Pescara, Chieti, Italy; ^3^CeSI-MeT, “G. d’Annunzio” University of Chieti-Pescara, Chieti, Italy; ^4^Synthesis and Solid State Pharmaceutical Centre (SSPC), School of Chemistry, University College Dublin, Dublin, Ireland; ^5^School of Chemistry, University of Nottingham, Nottingham, United Kingdom

**Keywords:** *Natrialba magadii*, *Haloferax volcanii*, extremozymes, glutathionyl-hydroquinone reductase, glutathione transferase, Xi class

## Abstract

Xi class glutathione transferases (GSTs) are a recently identified group, within this large superfamily of enzymes, specifically endowed with glutathione-dependent reductase activity on glutathionyl-hydroquinone. Enzymes belonging to this group are widely distributed in bacteria, fungi, and plants but not in higher eukaryotes. Xi class GSTs are also frequently found in archaea and here we focus on the enzyme produced by the extreme haloalkaliphilic archaeon *Natrialba magadii* (NmGHR). We investigated its function and stability and determined its 3D structure in the apo form by X-ray crystallography. NmGHR displays the same fold of its mesophilic counterparts, is enriched in negatively charged residues, which are evenly distributed along the surface of the protein, and is characterized by a peculiar distribution of hydrophobic residues. A distinctive feature of haloalkaliphilic archaea is their preference for γ-glutamyl-cysteine over glutathione as a reducing thiol. Indeed we found that the *N. magadii* genome lacks a gene coding for glutathione synthase. Analysis of NmGHR structure suggests that the thiol binding site (G-site) of the enzyme is well suited for hosting γ-glutamyl-cysteine.

## Introduction

Glutathione transferases (GSTs) constitute a superfamily of multifunctional proteins widely distributed in nature and they are found in both eukaryotic and prokaryotic organisms (Hayes et al., 2005; Allocati et al., 2009, 2012, 2014; Cummins et al., 2011; Wu and Dong, 2012). The main role of GSTs is the cellular detoxification of several xenobiotic and endogenous compounds, including drugs (Hayes et al., 2005; Allocati et al., 2018). They are also involved in other catalytic processes and are able to bind a number of molecules non-catalytically (Hayes et al., 2005). GSTs metabolize a wide variety of electrophilic compounds via glutathione (GSH) conjugation. GSH is the most abundant low molecular weight (LMW) thiol present in all eukaryotic cells, and it is involved in several physiological processes (Masip et al., 2006). In bacteria, it is detected in Gram-negative and in some Gram-positive species (Fahey, 2013). Eukaryotic GSTs are divided into at least three major families of proteins, namely soluble GSTs, mitochondrial GSTs and microsomal GSTs (Wu and Dong, 2012). Representatives of all three families are also present in bacteria (Allocati et al., 2009, 2012). In a previous work, analyzing the distribution of GSTs in Gram-positive bacteria and archaea, we found that the recently defined Xi class is the most represented in both groups (Meux et al., 2011). This class is also conserved in Gram-negative bacteria, fungi, protozoa and plants but not in animals (Belchik and Xun, 2011). Xi class GSTs form a distinct group of GST enzymes endowed with glutathionyl-hydroquinone reductase activity and show homology with the Omega class GSTs (Xun et al., 2010; Meux et al., 2011; Lallement et al., 2015). Omega class GSTs (GSTO) are known to exhibit a different range of enzymatic activities as compared to other GST classes, which were attributed to an N-terminal domain cysteine residue that can form a mixed disulfide bond with GSH (Board et al., 2000; Garcera et al., 2006). Similar to GSTOs, Xi class GSTs also contain a cysteine residue in their active site. This feature is also shared by GSTs belonging to other classes: Beta, Lambda, chloride intracellular channels (CLICs), and dehydroascorbate reductases (DHARs; Allocati et al., 2009; Schwartz et al., 2018).

The domain of Archaea represents a group of prokaryotes that can live in the most extreme environments on the planet such as the hottest or coldest habitats. Archaea are of great interest to biotechnology because their enzymes are able to catalyze reactions under extreme conditions. Hence, extremozymes are of key importance for the development of new biotechnological tools for sustainable development in a variety of industrial processes, including agricultural, chemical and pharmaceutical applications (Elleuche et al., 2014).

The present work describes the cloning, expression, purification, crystallization, and structure determination of NmGHR, a GST of the Xi class from the extreme haloalkaliphilic archaeon *Natrialba magadii* ATCC 43099. *N. magadii* – belonging to the class of *Halobacteria* – is an aerobic archaeon isolated from Lake Magadi in Kenya (Tindall et al., 1980) that optimally grows in 3.5 M NaCl, pH 9.5, and at a temperature range of 37 to 40°C. To the best of our knowledge, this is the first structure of a GST from a haloalkaliphilic archaeon.

## Materials and Methods

### Bacterial Strains and Growth Conditions

*N. magadii* ATCC 43099 strain was kindly provided by Rosana E. De Castro (Universidad Nacional de Mar del Plata, Argentina). *N. magadii* ATCC 43099 cells were grown at 37°C aerobically in Tindall’s modified medium containing yeast extract (5 g/L) as described previously (Gimenez et al., 2000).

*Escherichia coli* TOP10 was grown at 37°C in LB medium with 100 μg/mL ampicillin (One Shot TOP10 chemically competent cells, Invitrogen).

*Haloferax volcanii* H1325 and *Hfx. volcanii* His-tag vector pTA963 were generously provided by Thorsten Allers (University of Nottingham, United Kingdom) (Allers et al., 2010). *Hfx. volcanii* H1325 strain was grown at 42°C in Hv-YPC medium (Dyall-Smith, 2009).

### Bioinformatics Analysis

For multiple sequence alignments and phylogenetic analysis of Xi GSTs covering three domains of life, protein sequences from representative species were obtained from the NCBI database^1^. The entire genome sequence of *N. magadii* ATCC 43099 is currently available (Siddaramappa et al., 2012). Together with *N. magadii* genome other archaeal genomes^2^ were screened for the presence of GSTs and putative sequences retrieved were analyzed.

Sequence alignment was produced using Clustal Omega software^3^. The phylogenetic tree was constructed by the neighbor-joining method with MEGA 7.0 program (Kumar et al., 2016). The robustness of the branches was assessed by the bootstrap method with 1.000 replications. Only bootstrap values greater than 40% are shown.

### Cloning Strategy

Total RNA was extracted from *N. magadii* ATCC 43099 using an RNeasy Mini Kit (QIAGEN). Briefly, 10 mL of bacterial culture were pelletted and placed in 350 μL of lysis buffer and the manufacturer’s protocol was followed. The RNA was eluted in a volume of 60 μL of RNase-free water and quantified by measuring the absorbance at 260 nm. Purity was assessed by calculating the A260/A280 ratio and sample were immediately aliquoted and stored at –80°C.

Synthesis of cDNA was performed using the High-Capacity cDNA Reverse Transcription Kit (Thermo Fisher Scientific). Briefly, a mix containing 1 μg of RNA, 2 μL 10X RT Buffer, 0.8 μL of 25X dNTP mix (100 mM), 2 μL 10X Random Primers, 1 μL MultiScribe Reverse Transcriptase (50 U/μL), 1 μL RNase Inhibitor (100 μL) and RNase-free water to reach a final volume of 20 μL. The reaction was incubated at 25°C for 10 min, 37°C for 120 min, 85°C for 5 min and then at 4°C. cDNAs were stored at –20°C.

*nmagghr* cDNA was amplified by PCR using the following primers (BspHI and BamHI sites are underlined): Forw-BspHI, 5′-TTAATCATGAACATGCTCGTCGACGGCGAGTGG-3′, and Rev-BamHI, 5′-TATAGGATCCTCACCGACCTGCAGACGA-3′, both based on the published nucleotide sequence (accession gene number: NMAG_RS05605). The gene was amplified in a 30 μL reaction containing: 500 ng of cDNA, 0.5 μM of each primer, 0.8 μM dNTPs, 1.5 mM MgCl_2_ and 1.25 U of GoTaq Polymerase (Promega). Cycling conditions were: a hot-start at 95°C for 2 min, followed by 30 cycles of denaturation at 95°C for 30 s, annealing at 60°C for 30 s and extension at 72°C for 1 min and 5 s. A final extension at 72°C was employed for 5 min. Successful amplification was confirmed by agarose gel electrophoresis and the PCR product was first subcloned into pCR2.1-TOPO vector (TOPO TA Cloning Kit, Thermo Fisher Scientific) according to the manufacturer’s protocol and further sequencing to confirm a correct amplification. Then, the inserted fragment was digested with BspHI and BamHI (New England BioLabs) from pCR2.1 TOPO vector and inserted into the PciI and BamHI sites of pTA963 expression vector. Restriction products were visualized on a 0.8% agarose gel containing ethidium bromide (0.5 μg/mL). Appropriate bands were excised and extracted using Qiagen Gel extraction Kit. Ligations (10 μL) were performed using molar insert-to-vector ratios of 1:3 and 1 U of T4 DNA ligase (Promega) at 4°C overnight. The resulting plasmid (pTA963-*nmagghr*), was introduced into *E. coli* TOP10 ultracompetent cells (Invitrogen) by heat shock transformation and transformants were grown on LB agar and ampicillin. Positive colonies were inoculated into LB broth supplemented with ampicillin and grown overnight for plasmid extraction using Plasmid Miniprep Kit (Qiagen). Extracted plasmids were sent for bi-directional sequencing using primers: Forw-pTA963, 5′-ACCGATGCACACACCAGTC-3′, and Rev-pTA963, 5′-AAAGGGAACAAAAGCTGGAG-3′ to confirm successful ligation.

*Hfx. volcanii* H1325 competent cells were prepared and transformed with pTA963-*nmagghr* according to standard methods (Dyall-Smith, 2009). Positive transformants were selected by growth on Hv-YPC plates. Plates were incubated at 42°C for about 5 d, until colonies were visible, followed by further incubation at room temperature until colonies appear pink-pigmented.

### Expression of NmGHR in *Hfx. volcanii* H1325 and Purification of Recombinant Protein

For expression of the NmGHR protein, 50 mL of cell culture (grown for 48 h at 42°C with shaking at 200 rpm) was used to inoculate three 5-L flasks with 1 L of Hv-YPC broth and bacteria were incubated at 42°C with shaking at 200 rpm. The expression of recombinant protein was induced by the addition of L-tryptophan (5 mM) at the start of growth. After 48 h of incubation, archaeal cells were pelletted at 6.000× *g* for 10 min at 4°C. Cells were resuspended in 100 mM Tris–HCl, pH 7.5, 2 mM EDTA, 2 M NaCl buffer (buffer A) and disrupted by repeated sonication with a 6 mm microtip at 50% amplitude in 30 s intervals. The particulate material was removed by centrifugation at 18,000 rpm at 4°C for 1 h and the supernatant loaded onto a 1 mL His-trap FF column (GE Healthcare) equilibrated with buffer A. Fractions were pooled, concentrated by ultrafiltration, dialysed against 100 mM Tris–HCl, pH 7.5, 2 mM EDTA buffer with 3.5 M NaCl with Amicon ultra-15. Protein concentrations were determined by the Bradford method. The molecular size and homogeneity of the protein were examined by SDS-PAGE analysis. The molecular mass of the native enzyme was estimated using AKTAprime Plus equipped with Superdex 200 10/300 GL gel filtration column (GE Healthcare, Europe) using a mobile phase of 50 mM sodium phosphate buffer (pH 7.5) containing 3 M NaCl at a flow rate of 0.4 mL/min. The molecular weight markers were eluted in the same buffer. Blue Dextran 2000 was used to determine the column’s void volume.

### Enzyme Assays

GST activities with GSH or gamma-glutamylcysteine (γ-Glu-Cys) and 1-chloro-2,4-dinitrobenzene (CDNB) as second substrate were determined as previously described (Habig and Jakoby, 1981; Sugimoto et al., 1985), with modifications. Experiments were performed in 100 mM potassium buffer, pH 6.5, 3.42 M NaCl (or 3.42 M KCl), 1 mM DTT, 1 mM CDNB, 2 mM γ-Glu-Cys (or 2 mM GSH) and 100 μM of enzyme. GST activity toward ethacrynic acid was assayed by modifying the method of Habig and Jakoby (1981) as follow: 100 mM potassium buffer, pH 6.5, 3.42 M NaCl (or 3.42 M KCl), 1 mM DTT, 0.2 mM ethacrynic acid, 0.25 mM γ-Glu-Cys (or 0.25 mM GSH) and 100 μM of enzyme.

A modified procedure of Meux et al. (2011) for the measurement of S-glutathionyl-hydroquinone reductase activity with benzoquinone was carried out. Experiments were executed as follows: 1 mM 1,4-benzoquinone in 30 mM Tris–HCl, pH 8.0, and 3.42 M NaCl (or 3.42 M KCl), was used as baseline for an absorbance spectrum (230–400 nm). After 2 min, 1 mM GSH (or 1mM γ-Glu-Cys) was added to the reaction mixture, and the spectra were monitored every minute during 5 min. After, NmGHR was added in the reaction mixture and spectra were recorded every minute.

Thioltransferase activity was carried out using a modified version of the method (Holmgren and Aslund, 1995). Enzymatic activities were measured in 130 mM sodium phosphate, pH 6.8, 3.42 M NaCl (or 3.42 M KCl), 1 mM EDTA, 3 mM GSH or γ-Glu-Cys, and 0.3 mM dehydroascorbic acid (DHA).

The 2-hydroxyethyldisulfide (HED) assay was performed in the following conditions: 100 mM Tris–HCl, pH 8.0, 3.42 M NaCl (or 3.42 M KCl), 2 mM EDTA, 0.4 mM NADPH, 1mM GSH (or 1 mM γ-Glu-Cys), 0.1 mg/mL BSA, 6 μg/mL yeast glutathione reductase, and 0.7 mM HED.

All experiments were performed at different temperature ranging from 25 to 40°C.

### Circular Dichroism

Experiments were performed using a Jasco J710 apparatus (Jasco Inc., Easton, MD, United States) equipped with a Peltier apparatus for temperature control. Protein samples were 6 μM in 50 mM phosphate buffer, pH 7.4, supplemented with the indicated amount of NaCl or KCl (from 1.0 to 3.0 M). Spectra were collected in a 1 mm optical path length quartz cuvette (Hellma, Plainview, NY, United States) at 20°C, with a scanning speed of 100 nm/min. Spectra are the average of three accumulations. Thermal melt experiments were conducted by raising the temperature by 1°C/min, from 20°C and monitoring the signal at 210 nm.

### Crystallization and Data Collection

NmGHR crystallization conditions were screened using protein concentrated up to 6.6 mg/mL in 50 mM Tris–HCl pH 7.5, 3.5 M NaCl. Best diffracting crystal was obtained from the Index Screen (Hampton Research) using the Phoenix crystallization robot (Art Robbins). Crystals appeared at 21°C in a droplet formed by mixing equal volumes (0.5 μL) of the protein solution, and reservoir solution, containing polyethylene glycol (PEG) 3350 25% *w/v*, 0.1 M Tris–HCl pH 8.5 and 0.2 M lithium sulfate. Crystal was cryoprotected by increasing the PEG 3350 concentration up to 30% *w/v* before flash-freezing. Data were collected at the ID23-2 beamline of ESRF synchrotron (Grenoble, Fr), at a wavelength of 0.873 Å. Data were processed using XDS (Kabsch, 2010), Pointless and Aimless (Winn et al., 2011). Final statistics are reported in Table 1.

**Table 1:** Data collection and refinement statistics for NmGHR.

Data collection	
Beamline	ID23-2
Wavelength (Å)	0.873
Space group	*P*2_1_
Cell dimensions	*a* = 58.2 Å
	*b* = 62.3 Å
	*c* = 107.7 Å
	γ = 95.6°
Resolution range (Å)	53.6–2.61 (2.73–2.61)^a^
CC(1/2) (%)	98.1 (64.2)
Rmerge	0.29 (1.024)
Rpim	0.12 (0.41)
Mean *I/sigma I*	5.9 (2.0)
Completeness (%)	100 (99.7)
Reflections	
Total no. observed	159010
Average multiplicity	6.8 (7.1)
Wilson plot *B* value	33.8
**Refinement statistics**	
Resolution range (Å)	53.6–2.61
R_work_/R_free_	21.4/26.1
Average B-factor (Å^2^) (no. of atoms)	
Protein	35.0 (5352)
Waters	29.2 (169)
RMSD	
Bond length (Å)	0.003
Bond angle (°)	0.7
Ramachandran (*n*,%)	
Favored	94.53
Allowed	5.16
Outliers	0.31

*^*a*^Values in parentheses refer to the highest resolution shell.*

### Structure Determination and Refinement

Data were phased by molecular replacement using the monomeric putative GST from *Corynebacterium glutamicum* (PDB code 3M1G) as a search model in PHASER (McCoy et al., 2007) as implemented in the PHENIX suite (Adams et al., 2010). Cycles of model building and refinement were carried out using Coot (Emsley and Cowtan, 2004) and Phenix.refine (Afonine et al., 2012). Water molecules were added by ARP/wARP (Lamzin et al., 2001) and visually inspected. The final model consists of two molecules (one dimer) per asymmetric unit. All the residues for the chain A were fitted into the electron density (including the N-terminal His-tag) with the exception of the last 14 residues. No electron density was observed for residues 14–22 as well as for the last 11 residues of chain B. Final statistics are reported in Table 1.

Figures were prepared using PyMOL Molecular Graphics System (The PyMOL Molecular Graphics System, Version 2.0 Schrödinger, LLC).

Coordinates and structure factors were deposited in the Protein Data Bank^4^ with accession code: 6GZF.

## Results and Discussion

### Analysis of NmGHR

Screening of fully sequenced *N. magadii* genome revealed only one gene coding for GHR (NmGHR). We also analyzed other representative archaeal genomes (see 2012 Biochimie) and found the presence of a single gene like in *N. magadii*. Only in two cases (*Halogeometricum borinquense* DMS 11551 and *Halorubrum lacusprofundi* ATCC 49239) two genes coding for putative GHR isozymes were detected.

**FIGURE 1:**
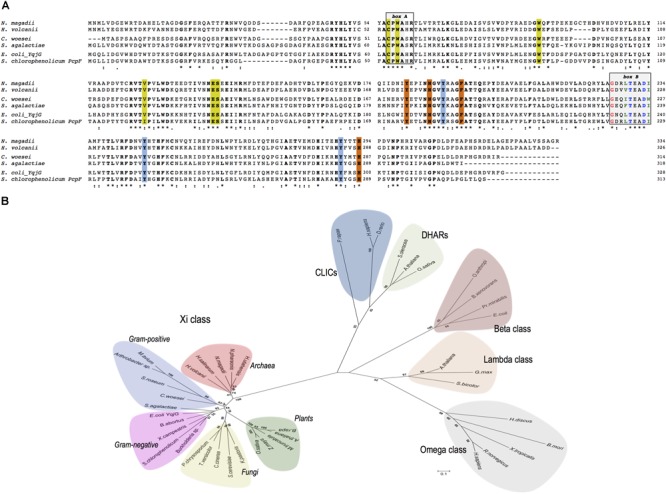
**(A)** Multiple sequences alignment of representative prokaryotic Xi class GSTs of *Haloferax volcanii* DS2, *Conexibacter woesei, Streptococcus agalactiae, Escherichia coli* YqjG and *Sphingobium chlorophenolicum* PcpF in comparison with *Natrialba magadii* ATCC 43099. **Box A**: strictly conserved motif. **Box B**: The N-capping box and the hydrophobic staple motif residues are shown in blue and green, respectively; the conserved glycine residue is shown in red. Catalytic residues are indicated in yellow, while the tyrosine triad typical of Xi class GSTs are indicated in cyano. Other H-site residues are indicated in orange. All conserved residues are shown in bold. (^∗^), identical residue; (:), conserved substitution; (.), semi conserved substitution. **(B)** Evolutionary relationships among prokaryotic and eukaryotic Xi GSTs and representative members of Omega, Beta, and Lambda GST classes as well as DHARs and CLIC proteins. The scale bar represents a distance of 0.1 substitutions per site. The sequences have the following accession numbers: **Xi class**, Archaea: *N. magadii* ATCC 43099 (WP_004217051), *Hfx. volcanii* DS2 (WP_004044265), *Halobacterium salinarum* (Q9HN26), *Halorhabdus utahensis* (WP_015790270), *Natronomonas pharaonis* (Q3IMP3); Gram-positive: *S. agalactiae* (Q8E0D4), *Arthrobacter* sp. (WP_011691995), *Mycobacterium avium* (WP_011723887), *C. woesei* (WP_012936861), *Streptosporangium roseum* (WP_012894656); Gram-negative: *E. coli* YqjG (P42620), *S. chlorophenolicum* PcpF (Q8KN33), *Xanthomonas campestris* (Q8P4Y7), *Brucella abortus* (WP_002964665), *Burkholderia* sp. (WP_008353845), Fungi: *Phanerochaete chrysosporium* (ACF15453), *Saccharomyces cerevisiae* (P36156), *Komagataella pastoris* (ANZ75071), *Coprinopsis cinerea* (XP_001836803), *Trametes versicolor* (Tv66368); Plants: *Arabidopsis thaliana* (Q9FL95), *Medicago truncatula* (Q2MGR6), *Brassica rapa* (XP_009123615), *Oryza sativa* (Q6EQX0), *Zea mays* (ACG32999); **Omega class**: *Bombyx mori* (AB365127), *Homo sapiens* (NP_004823), *Rattus norvegicus* (Q9Z339), *Xenopus tropicalis* (Q5PPQ3), *Haliotis discus* (ABO26600); **Beta class:** *Proteus mirabilis* (P15214), *E. coli* (P39100), *Ochrobactrum anthropi* (P81065), *Burkholderia xenovorans* LB400 (Q9RAFO); **Lambda class:** *A. thaliana* (Q6NLB0), *Sorghum bicolor* (A0A1B6P694), *Glycine max* (I1L8Q0); **DHARs**: *Spinacia oleracea* (Q9FVE4-1), *A. thaliana* (Q9FWR4), *O. sativa* (Q65XA0); **CLICs**: *H. sapiens* (Q5SRT3), *Pieris rapae* (A0A220K8N3*), Danio rerio* (Q6NYF2).

NmGHR (accession number: WP_004217051) consists of 334 amino acids and shares the same domain structure of all GSTs analyzed so far. Multiple alignment analysis of NmGHR in comparison with other Xi class GSTs showed that all sequences display several common motifs (Figure 1A).

All members exhibit a strictly conserved CPWA motif that could play a key role in the binding of GS-(hydro)-quinones (Figure 1A, box A) (Green et al., 2012; Lallement et al., 2015; Schwartz et al., 2016). This motif is also compatible with the CP(W/F/Y)(A/S)(H/Q)R motif found in GSTOs, with the exception that the (H/Q) residue may be replaced by a serine (Figure 1A) (Meux et al., 2011). The first Cys residue of this motif is thought to play a fundamental role in the catalytic cycle of GSTOs (Board et al., 2000; Yamamoto et al., 2013). It is located in the GSH binding domain (G-site) of GSTOs and is involved in the glutathionylation and deglutathionylation activities shown by these enzymes. The following conserved Pro residue is thought to promote optimal positioning of the Cys thiol for the stabilization of the thiolate form (Board et al., 2000). Like other cytosolic GSTs, including GSTOs, all members of the Xi class possess two highly conserved structural motifs in the H-site, an N-capping box and a hydrophobic motif involved in protein folding and stability (Figure 1A, box B) (Allocati et al., 2006). An additional strictly conserved glycine residue located four amino acids before the N-cap residue is also present. Other characteristic residues which have been shown to bind GSH are also conserved in all sequences suggesting a similar role in the enzymes (Figure 1A).

A phylogenetic analysis using representative members of prokaryotic and eukaryotic Xi class GSTs and their homologous belonging to different GST classes characterized by the presence of a cysteine residue in the G-site, is shown in Figure 1B. This analysis suggests that Xi class GSTs are closely related and form a distinct group that includes gram-positive and gram negative bacteria, archaea but also plants and fungi. It is also of interest to note that bacterial beta class GSTs, which display canonical transferase activity, are more closely related to the eukaryotic lambda and omega classes than to gram-positive and gram-negative Xi class GSTs.

### Expression of NmGHR

**FIGURE 2:**
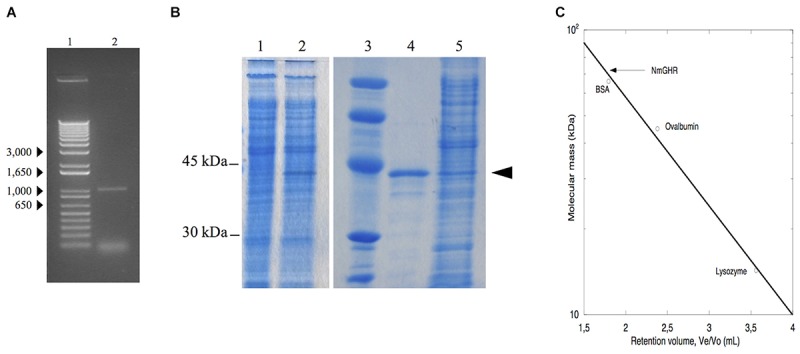
Analysis of NmGHR. **(A)** PCR amplified *nmagghr* gene. Lane 1, molecular weight markers; lane 2, *nmagghr*. **(B)** SDS/PAGE analysis of total cellular extracts of *Hfx. volcanii* H1325 bearing pTA963-nmagghr. Lane 1, crude extract before induction; lanes 2 and 5, crude extract after induction; lane 3, molecular mass markers; lane 4, Ni-affinity purified cloned NmGHR showed by the arrowhead. **(C)** molecular mass of the native enzyme estimated by gel filtration chromatography. Molecular weight markers (kDa): bovine serum albumin (BSA), 66; ovalbumin, 45; lysozyme, 14.3. V_e_: elution volume, V_o_: void volume.

Although it appears that *nmagghr* gene does not contain introns, we synthesized cDNA from the total RNA extracted from *N. magadii* ATCC 43099 strain first of all to verify whether the mRNA for this gene was effectively transcribed under the growth conditions adopted in our laboratory. The cDNA was amplified by PCR using primers designed on the nucleotide sequence of the *nmagghr* gene. The length of the transcribed gene was about 1000 bp, in agreement with the dimension of the gene (1005 bp) (Figure 2A).

In a first attempt to obtain the protein in quantities amenable for structural and functional characterization, the *nmagghr* cDNA was cloned into a pET28a(+) vector for expression of a N-terminal 6xHis-tagged protein and expressed in *E. coli*. NmGHR was indeed expressed as soluble protein, as detected from SDS-PAGE of crude lysates, but it was lost during purification. We reasoned that the absence of high salt concentrations typical of the halophilic host might influence protein fold or stability, resulting in aggregation and precipitation (Madern et al., 2000). For this reason we decided to switch to an expression system more similar to the growing conditions of the native host, i.e., *Hfx. volcanii*, which is widely used for haloarchaeal genetics (Albers and Meyer, 2011) and has also recently been developed as a system for protein overexpression (Allers et al., 2010). *Hfx. volcanii* H1325 cells, transformed with the pTA963 plasmid bearing the *nmagghr* gene overexpressed a protein, tagged at the N-terminus with a non-cleavable hexahistidine sequence, with an apparent subunit molecular mass of 38 kDa on SDS-PAGE (Figure 2B) and a relative molecular mass of 77 kDa on gel filtration column (Figure 2C) suggesting that it acts as a dimer in solution.

### Circular Dichroism

**FIGURE 3:**
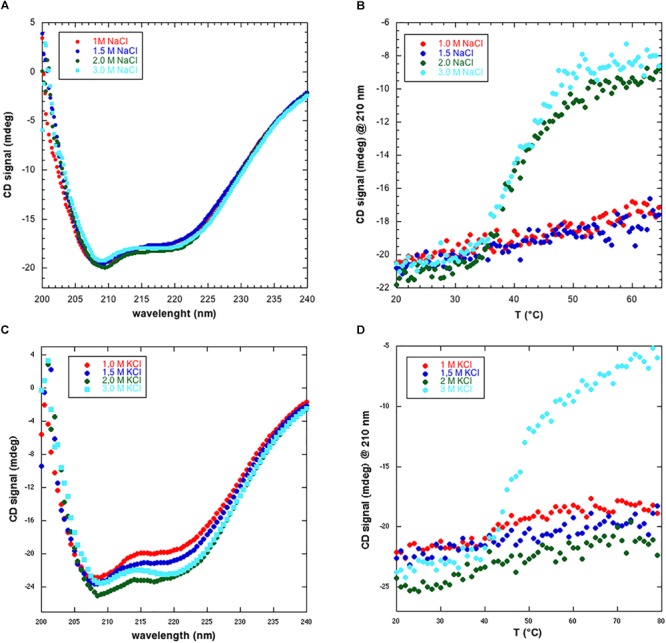
Circular dichroism analysis of NmGHR. **(A)** Static spectra indicate a folded and predominantly α-helical protein at all NaCl concentrations tested. **(B)** Thermal melt analysis reveals a cooperative denaturation profile at 2.0 and 3.0 M NaCl. Conversely, at 1.0 and 1.5 M NaCl, signal only slightly and linearly increases as a function of temperature, indicating resistance to denaturation possibly due to aggregation at these lower ionic strength conditions. **(C)** Static spectra of NmGHR in the presence of the indicated amount of KCl. **(D)** Thermal melt analysis in the presence of different concentrations of KCl. A cooperative but incomplete denaturation profile is observed only in the presence of 3.0 M KCl.

To gain a first hint into the structure and stability of NmGHR we collected CD spectra in four different salt concentrations, i.e., 1.0, 1.5, 2.0, and 3.0 M NaCl. Static CD spectra, shown in Figure 3A, highlight the presence of a folded protein with predominantly α-helical structure in all conditions. However, thermal melt analysis of the protein, in the same conditions, highlighted some differences. In fact, as shown in Figure 3B, the protein shows a cooperative denaturation profile only in the presence of 2.0 and 3.0 M NaCl. Conversely, at the lower salt amounts of 1.0 and 1.5 M NaCl, the signal at 210 nm only slightly increases when raising the temperature from 20 to 65°C. To account for possible effects of different cations, the same experiments were repeated replacing NaCl with KCl. Also in this case, static CD spectra indicate the presence of folded protein in all conditions (Figure 3C). A different thermal melt behavior was instead observed. In fact, contrary to what happens with NaCl (Figure 3B), the protein is still resistant to thermal denaturation at 2.0 M KCl while it denatures at 3.0 M NaCl (Figure 3D). The denaturation profile is slightly different to what observed with NaCl. In fact, while with 3.0 M NaCl a plateau is reached at 65°C, in the case of KCl the protein is still not fully denatured at 80°C and the midpoint of denaturation (Tm) is placed at higher temperature. These data suggest that different cations may differently influence protein stability. Collectively, we hypothesize that at lower salt amounts, the protein is correctly folded, as suggested by static CD spectra, but may undergo aggregation and be more resistant to thermal denaturation. Furthermore, CD data suggest that NmGHR is monodisperse at 3.0 M NaCl or KCl and that further functional and structural studies should be performed in these conditions.

### Enzymatic Activity

GSH is produced through two sequential enzymatic reactions catalyzed by glutamate-cysteine ligase (*gsh*A) and GSH synthetase (*gsh*B). In some bacteria, GSH is produced by a bifunctional enzyme (GshF) catalyzing both reactions simultaneously (Yang et al., 2016). Prokaryotes characterized by the absence of GSH, produce other LMW thiols – such as mycothiol, bacillithiol and CoA – with a similar protective role to GSH (Fahey, 2013). GSH is also absent in Archaea (Fahey, 2013). In halobacteria, γ-Glu-Cys is the predominant thiol, which is more stable in high salt concentrations than GSH (Newton and Javor, 1985; Fahey, 2013). Consistently, our analysis of the genome sequence of *N. magadii* ATCC 43099 indicated the presence of a *gshA* gene encoding a glutamate-cysteine ligase (Accession number: WP_004214852) but neither *gshB* nor *gshF* genes.

Several enzymatic activities played by NmGHR were tested using γ-Glu-Cys as a co-substrate. The main activity of Xi class GSTs is thought to be the S-glutathionyl-hydroquinone thiol-dependent reduction and this was monitored using benzoquinone as a model substrate. Classic conjugation activity was monitored using 1-chloro-2,4-dinitrobenzene (CDNB) and ethacrynic acid as second substrates. Thioltransferase activity with DHA and HED were also tested. In all cases, we could not detect any activity with γ-Glu-Cys as a thiol nor with GSH that we tested as a negative control. All experiments were performed in 3.42 M NaCl and at temperatures ranging from 25 to 40°C. Same experiments were also repeated by using 3.42 M KCl instead of NaCl; also in this case we could not detect any activity. The lack of enzymatic activity that we observed may not be explained by the absence in the primary structure of residues necessary for catalysis, as shown in Figure 1A. Conversely, it is possible that the model substrates that we used are not recognized by this particular enzyme which may preferentially work on different substrates which are presently unknown.

To gain further insight, we decided to determine the structure of the protein by X-ray crystallography.

### NmGHR Overall Structure

**FIGURE 4:**
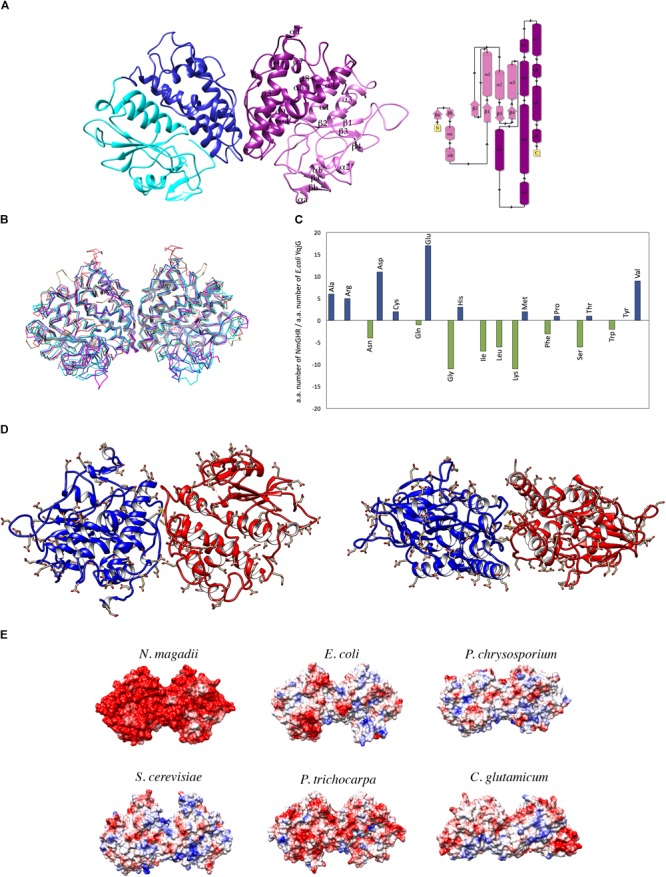
Structural analysis of NmGHR. **(A)** Crystal structure of NmGHR physiological dimer. Thioredoxin-like domains are represented in cyan and pink, while C-terminal α-helical domains are represented in blue and magenta, respectively. A topology diagram indicating secondary structure elements and their numbering is also shown. **(B)** Structural superposition of NmGHR (blue) and the Xi class GSTs from *E. coli* (EcYqjG, in cyan), *C. glutamicum* (brown), *S. cerevisiae* (orange), *P. trichocarpa* (magenta) and *P. chrysosporium* (green). RMSD values are reported in Table 2. **(C)** Amino acid composition differences between NmGHR and EcYqjG. Blue and green bars indicate, respectively, the increase or decrease in the amount of a given NmGHR amino acid with respect to the same in EcYqjG. **(D)** Frontal and up view of NmGHR with negatively charged residues represented in sticks. Aspartate and glutamates are evenly distributed all over the surface of the protein. **(E)** Electrostatic potential surface of NmGHR in comparison with those of representative Xi class GSTs from mesophilic organisms (same as in panel B). Red stands for negative charge, blue for positive charge.

NmGHR was crystallized under high salt conditions and diffraction data were collected to 2.6 Å resolution. The structure was solved by molecular replacement using the structure of the GST from *C. glutamicum* (PDB code 3M1G) as a model. Statistics about data collection, processing and refinement are reported in Table 1. The physiological dimeric assembly of NmGHR is shown in Figure 4A together with a topology diagram showing secondary structure elements. The NmGHR protein backbone was superposed to the structures of Xi class GSTs from *E. coli*, also known as EcYqjG, *C. glutamicum, Saccharomyces cerevisiae, Phanerochaete chrysosporium*, and *Populus trichocarpa*, as shown in Figure 4B. NmGHR displays the canonical GST fold with a N-terminal thioredoxin-like domain and a C-terminal all-helical domain. The protein backbone and relative orientation of the monomers in the dimeric assembly is remarkably well conserved between archaeal NmGHR and it mesophilic counterparts. Indeed, data reported in Table 2 indicate that, when superposing the structure of NmGHR with those of homologs belonging to gram-positive, gram-negative, yeast, fungal or plant organisms, the root mean square deviations (RMSD) between equivalent Cα are comprised between 1.1 and 1.3 Å. Furthermore these values are comprised between 1.2 and 1.78 Å when directly superposing the dimers (Table 2). These observations suggests that not only the canonical GST fold is perfectly compatible with halotolerance but also the quaternary structure, posing this class of enzymes as one of the most structurally conserved when comparing halophilic proteins to their mesophilic counterparts (Graziano and Merlino, 2014).

**Table 2:** Average root mean square deviations (RMSD) of equivalent Cα in the superposition between NmGHR and representative Xi class enzymes from mesophilic organisms.

PDB	RMSD Monomer (Å) (n. aligned residues)	RMSD Dimer (Å) (n. aligned residues)	Sequence identity (%)	Color in Figure 4B
4GOI (*E. coli*)	1.31 (306)	1.78 (604)	51.32	Cyan
3PPU (*P. chrysosporium*)	1.19 (279)	1.54 (556)	48.20	Green
5LKB (*S. cerevisiae*)	1.1 (271)	1.26 (545)	42.75	Orange
4USS (*P. trichocarpa*)	1.05 (292)	1.21 (584)	47.26	Magenta
3M1G (*C. glutamicum*)	1.24 (241)	1.53 (486)	44.65	Brown

Main differences are therefore limited to the enrichment and distribution of charged, polar and apolar residues, with a particular contribution played by negatively charged residues. Indeed as shown in Figure 4C, aspartate and glutamate residues in NmGHR outnumber the corresponding ones in EcYqjG by 12 and 17 residues, respectively. Moreover a markedly reduced content of lysine residues is present which is not balanced by the slightly higher content of arginines. The amount of hydrophobic residues is also reduced in NmGHR with respect to EcYqjG; within this group of residues it may also be noted a strong preference in NmGHR for valines and alanines with respect to isoleucines and leucines. Another peculiar feature is the consistently reduced number of glycine residues in NmGHR with respect to EcYqjG (Figure 4C).

As shown in Figure 4D, negatively charged residues are evenly distributed in the surface of the protein, both at the N-terminal thioredoxin and C-terminal all-helical domain. This is consistent with the main features observed in other halophilic proteins which must adapt to high saline concentrations (Madern et al., 2000) and is also highlighted in Figure 4E, where the electrostatic surface of NmGHR is represented in comparison with those of its mesophilic homologs (same as in Figure 4B). The molecular basis of halotolerance have not been fully understood yet (Graziano and Merlino, 2014), however, it has been proposed that negatively charged residues are required to help the maintenance of protein conformational stability (Madern et al., 2000). Alternatively, a role for a strong negatively charged surface in preventing protein aggregation has been proposed (Elcock and McCammon, 1998). This is consistent with our CD data suggesting that by reducing the ionic strength, NmGHR tends to aggregate (Figures 3B,D).

**FIGURE 5:**
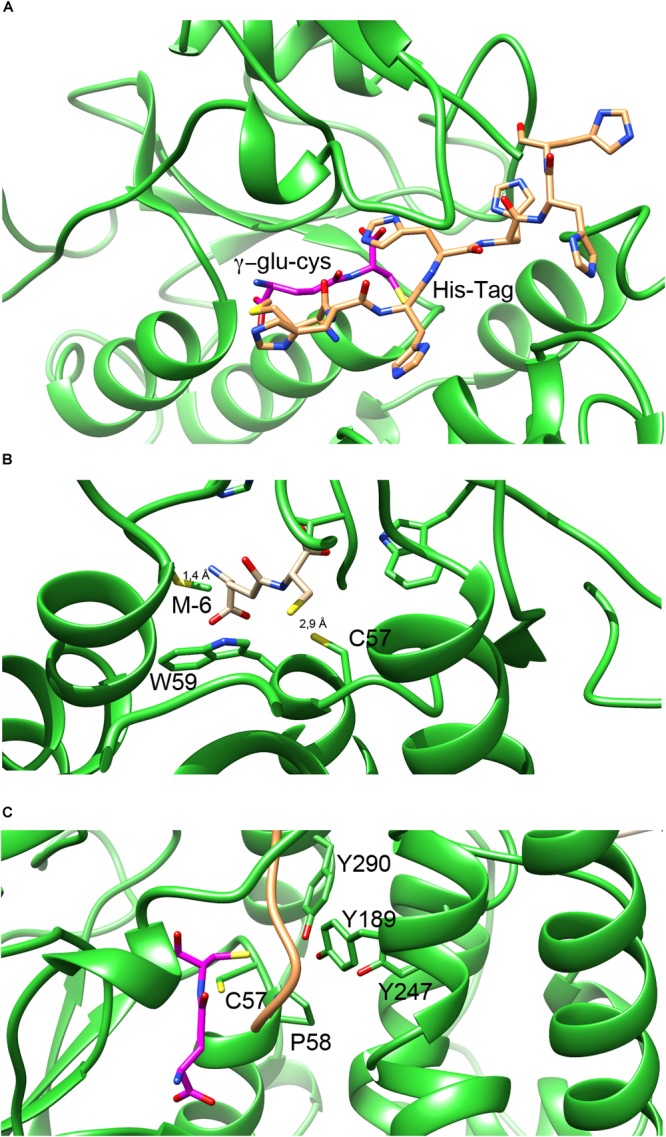
G- and H-sites in NmGHR. **(A)** The hexa-histidine tag, represented in orange sticks occupies a deep cleft in between the N-terminal thioredoxin domain and the C-terminal α-helical domain. Deeper inside the structure, the G-site is marked by the modeled γ-Glu-Cys (represented in magenta sticks). **(B)** Close-up view of γ-Glu-Cys in the G-site of the protein. The position of γ-Glu-Cys was modeled based on the position of GSH in the superimposed structure of EcYqjG. It may be noted that Cys57 is conveniently located at 2.9 Å distance from the γ-Glu-Cys sulfhydryl group. However, the N-terminal amino group of modeled γ-Glu-Cys is only 1.4 Å apart from the Met(-6) residue, indicating that the hexa-histidine tag would interfere with γ- Glu-Cys binding, under these experimental conditions. **(C)** The position of the H-site is highlighted by the tyrosine triad (Tyr189, Tyr247, and Tyr290) that were shown with EcYqjG to be implicated in substrate binding. The hexa-histidine tag, shown in orange ribbon, sits in the proximity of the three tyrosines, occupying the position where the substrates bind.

An interesting feature of NmGHR structure is that the histidine-tag present at the N-terminus of the protein chain to aid purification is ordered and visible. We could model in the electron density the complete tag including the initial methionine of chain A, and almost completely also of chain B. As shown in Figure 5A, the tag sits along a wide cleft between the N-terminal and C-terminal domains of NmGHR. This is also the place in GSTs where the co-substrate H-site is located (see below). The histidine tag is usually very flexible and not visible in electron density maps. However, it is well known that high ionic strength induces structural compactness in proteins and therefore the high salt conditions in which the protein was purified and crystallized may have facilitated the compaction of the histidine tag onto the surface of the protein.

### NmGHR G- and H- Sites

One of the main features of *N. magadii* and other halophiles is their inability to synthesize GSH and the preferential use of γ-Glu-Cys as a reducing thiol. For this reason we performed both co-crystallization and soaking experiments with γ-Glu-Cys. Unfortunately, despite many attempts, we could never detect electron density convincingly accounting for the thiol in our maps. Therefore, to visualize the putative γ-Glu-Cys molecule bound to the NmGHR site we adopted an indirect modeling approach. We superposed NmGHR and EcYqjG structures and positioned the GSH molecule of EcYqjG in the same position in the structure of NmGHR; then we removed the glycine moiety of GSH to yield a γ-Glu-Cys molecule. The result of this procedure is shown in Figure 5. It may be noted that the NmGHR Cys57 sulfhydryl group is located at 2.9 Å from the sulfhydryl group of the modeled γ-Glu-Cys, suggesting that the γ-Glu-Cys in this position might be functionally competent (Figure 5B). However, it may also be noted that, under this framework, the amino-terminal group of γ-Glu-Cys is as close as 1.4 Å from the side chain of the methionine (-6) that precedes the hexa-histidine-tag (Figure 5B). This suggests that, in our experimental conditions, the histidine-tag partly occludes the enzyme’s G-site, possibly explaining why our soaking or co-crystallization experiments failed to recover γ-Glu-Cys in the crystals. Figure 5C highlights the position of G-site residues (Cys57 and the invariant cis Pro58) and H-site residues. In particular, three tyrosine residues (Tyr189, Tyr247, and Tyr290) are perfectly conserved in all Xi class GSTs and have been shown in EcYqjG to play a fundamental role in substrate binding. It may be noted that the hexahistidine tag, represented as an orange ribbon, is placed exactly in between Cys57 and γ-Glu-Cys, that occupy the G-site, and the tyrosine triad that shape the H-site. From this analysis it appears that the position that would be engaged by the substrates in the catalytic cycle of GHRs is actually occupied by the hexa-histidine tag in our structure. Therefore the absence of S-glutathionyl-hydroquinone reductase activity that we detected with the classical substrate for Xi class GSTs, i.e., benzoquinone, might be due to the position occupied by the tag in high salt conditions. A detrimental effect of the histidine-tag on enzymatic activity has previously been observed in other cases (Sabaty et al., 2013).

## Conclusion

In this work, we expressed and purified for the first time the Xi class GST from *N. magadii*, a haloalkaliphilic archaeon living in extremely harsh conditions. Structural analysis suggested that the protein is stable and monodisperse in high salt while tends to aggregate with decreasing ionic strength. A comparison of the crystal structure of NmGHR with its mesophilic counterpart from *E. coli*, suggested that the overall protein fold is perfectly conserved. As expected, NmGHR is characterized by an extremely high content of negatively charged residues which are evenly distributed throughout the surface of the protein. Marked differences in the amounts of various hydrophobic residues were also observed. Analysis of the active site suggested conservation of residues implicated in γ-Glu-Cys binding and shaping the H-site. However, the position of the hexa-histidine tag, which sits in between the G- and H-site of the protein, prevented us from direct experimental confirmation of enzymatic activity.

## Author Contributions

NA conceived and designed the study, analyzed the data and wrote the paper. LF analyzed the data and wrote the paper. ADM, MM, EC, DS, and JC performed the experiments. CDI and FP analyzed the data and critically read the manuscript before submission.

## Conflict of Interest Statement

The authors declare that the research was conducted in the absence of any commercial or financial relationships that could be construed as a potential conflict of interest.

## Footnotes

1 http://www.ncbi.nlm.nih.gov/protein/

2 http://www.ncbi.nlm.nih.gov/genome/

3 www.ebi.ac.uk/Tools/msa/clustalo

4 https://www.rcsb.org
